# Ubiquitous Graphene Electronics on Scotch Tape

**DOI:** 10.1038/srep12575

**Published:** 2015-07-29

**Authors:** Yoonyoung Chung, Hyun Ho Kim, Sangryun Lee, Eunho Lee, Seong Won Kim, Seunghwa Ryu, Kilwon Cho

**Affiliations:** 1 Department of Electrical Engineering, Pohang University of Science and Technology, Pohang 37073, Korea; 2 Department of Chemical Engineering, Pohang University of Science and Technology, Pohang 37073, Korea; 3 Department of Mechanical Engineering, Korea. Advanced Institute of Science and Technology, Daejeon 34141, Korea

## Abstract

We report a novel concept of graphene transistors on Scotch tape for use in ubiquitous electronic systems. Unlike common plastic substrates such as polyimide and polyethylene terephthalate, the Scotch tape substrate is easily attached onto various objects such as banknotes, curved surfaces, and human skin, which implies potential applications wherein electronics can be placed in any desired position. Furthermore, the soft Scotch tape serves as an attractive substrate for flexible/foldable electronics that can be significantly bent, or even crumpled. We found that the adhesive layer of the tape with a relatively low shear modulus relaxes the strain when subjected to bending. The capacitance of the gate dielectric made of oxidized aluminum oxide was 1.5 μF cm^−2^, so that a supply voltage of only 2.5 V was sufficient to operate the devices. As-fabricated graphene transistors on Scotch tape exhibited high electron mobility of 1326 (±155) cm^2^ V^−1^ s^−1^; the transistors still showed high mobility of 1254 (±478) cm^2^ V^−1^ s^−1^ even after they were crumpled.

Recent advances in flexible device technology have changed the paradigm of electronics from rigid objects to flexible form factors^1^. Flexible displays^2^, sensors^3^,^4^, circuits^5^,^6^, solar cells^7^, and batteries^8^ have become popular subjects in both academia and industry, and such components have facilitated the development of bendable consumer products. One of the most popular approaches for making flexible devices is to use flexible plastic substrates, such as polyimide and polyethylene terephthalate, and to fabricate electronic components on them at a low temperature at which substrate deformation does not occur^9^. Alternatively, flexible and conformable substrates such as polydimethylsiloxane and a commercial tattoo can also be used on which a thin layer of electronic devices is laminated^5^,^10^. Flexible devices can also be directly prepared onto nonconventional substrates including banknotes and papers^11^,^12^,^13^. These methods have gradually expanded the use of electronics.

In this study, we developed a method for flexible and conformable electronic devices on Scotch tape. Scotch tape, which serves as a remarkably flexible substrate, is simply attached on various objects as long as the adhesive of the tape adheres; as a result, Scotch tape electronics can be utilized to accomplish ubiquitous electronic systems for applications of interest. Another advantage of such a Scotch tape substrate is that the adhesive layer of the tape relaxes the strain when subjected to bending. We analyzed the mechanical strain applied to a bent Scotch tape substrate. Because of its soft layers, transistors on top of the Scotch tape, i.e., non-adhesive side, experience a significantly lower tensile strain as compared to regular polyimide under the same bending condition. We used graphene as the channel material. Graphene is not only one of the most representative flexible electronic materials^14^,^15^, but also a promising candidate for signal conditioning in electronics^16^,^17^. In particular, graphene devices are widely studied for the radio-frequency technology, which enables wireless communication^18^,^19^. Thus, the fabrication of graphene transistors on Scotch tape would be a significant step toward realizing ubiquitous electronics.

The device structure is shown in Fig. 1. Graphene field-effect transistors (GFETs) were fabricated on Scotch tape attached on a silicon dioxide (SiO_2_) wafer (Fig. 1a). After fabrication, the Scotch tape substrate was peeled off from the wafer and ready to be placed onto various nonconventional surfaces (Fig. 1b). As shown in Fig. 1c, the GFETs on the Scotch tape were easily attached on a banknote, human skin, and a pen without any adverse effects on the electrical properties. Scotch tape as a substrate is superior to widely used polyimide for flexible/foldable electronics; the Young’s modulus of the Scotch tape in this study is approximately 0.4 GPa as compared to 2.5 GPa for polyimide^20^. However, Scotch tape exhibits a lower operating temperature, less chemical resistance, and more outgassing in a vacuum chamber than polyimide, thereby requiring mild fabrication procedures to prevent any defects in the delicate substrate.

The gate dielectric is one of the most important components in field-effect transistors (FETs). Especially, high capacitance with a low leakage current is essential for low-power electronic devices that can be operated by portable batteries. Several methods have been used to meet the dielectric requirements for flexible transistors: atomic layer deposition (ALD) of aluminum oxide (AlO_X_)^21^,^22^, ion-gel dielectrics^23^,^24^, and oxidation of aluminum thin films^25^. Among these approaches, the oxidation of aluminum thin films with oxygen plasma, which does not require high temperature, solvent, or high-purity gas, is a feasible process for a Scotch tape substrate. Previously, oxygen plasma treatment on aluminum was demonstrated to form a high-capacitance AlO_X_ gate dielectric for flexible organic transistors; however, an additional self-assembled monolayer (SAM) prepared by soaking in a solution was required on AlO_X_ or else the leakage current through the dielectric increased significantly^25^. As solution-processed SAMs are not suitable for Scotch tape substrates, we improved the fabrication process for oxidized AlO_X_ by optimizing the plasma conditions so that a single AlO_X_ layer could be used as the gate dielectric for GFETs.

In a plasma system, the amount and movement of plasma particles are affected by power and pressure^26^. In particular, the kinetic energy of plasma ions is related to pressure, as shown in Equation 1, which describes the mean free path of a gaseous particle


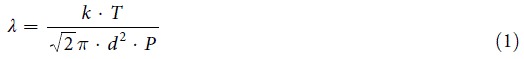


where *k* is the Boltzmann constant, *T* is the temperature, *d* is the collision diameter of the molecule, and *P* is the pressure. Therefore, oxygen radicals can penetrate the aluminum surface more effectively with a higher kinetic energy under lower pressure. We oxidized the surface of aluminum thin film (30 nm) deposited on a SiO_2_ wafer in an oxygen plasma chamber for 7 min. During plasma treatment, three different oxygen pressure values were used: 12, 50, and 100 mTorr. The electrical properties of the oxidized AlO_X_ were tested after the deposition of the gold top electrode. The AlO_X_ samples prepared using different oxygen pressures had an almost identical capacitance of 1.5 (±0.1) μF cm^−2^, which is equivalent to the capacitance of 2.3-nm-thick SiO_2_. However, there was a considerable difference in the leakage current of the three AlO_X_ samples. As the oxygen pressure for plasma treatment decreased, the leakage current through the AlO_X_ layer decreased, and the breakdown voltage increased (Fig. 2a). These results indicate that lower oxygen pressure facilitates a denser oxide film with better quality. Because of the large capacitance, only 1–1.5 V is sufficient to operate FETs by using the plasma-treated AlO_X_ as the gate dielectric. In a previous study in which the ALD of AlO_X_ with a SAM was employed^27^, the capacitance of the gate dielectric (total thickness of 6.4 nm) was approximately 0.5 μF cm^−2^, and its leakage current was on the order of 10^−7^ A cm^−2^ at 3 V. Applying 3 V to the 0.5 μF cm^−2^ capacitor induces an amount of charges equal to that from our 1.5 μF cm^−2^ AlO_X_ capacitor with only 1 V. In Fig. 2a, the gate leakage current through AlO_X_ (12 mTorr oxygen) at 1 V is on the order of 10^−8^ A cm^−2^, an order of magnitude lower than that in the previous study. We performed X-ray photoelectron spectroscopy (XPS) on the AlO_X_ samples to investigate their chemical composition. The inelastic mean free path of electrons in AlO_X_ is less than 5 nm at 1420 eV—the maximum electron kinetic energy in this XPS measurement^28^. Therefore, the angle between the photoelectron detector and the sample stage was tilted by 80° to ensure that all of the measured photoelectrons came from AlO_X_. The atomic ratio of AlO_X_ as a function of oxygen pressure is shown in Fig. 2b; lower oxygen pressure during the plasma resulted in a higher relative ratio of oxygen. (Note that the oxygen ratio was overestimated to some degree because oxygen and water molecules adsorbed on the AlO_X_ surface cannot be completely removed even under high-vacuum conditions.) Thus, the insulating property of AlO_X_ is improved with the increase in the oxygen ratio. The thickness of AlO_X_ fabricated at an oxygen pressure of 12 mTorr was measured to be 5.7 nm by transmission electron spectroscopy (TEM), as shown in the inset of Fig. 2b. By assuming a dielectric constant of 9.5 for the AlO_X_ layer, the measured thickness is in good agreement with the capacitance value above.

We fabricated GFETs on a Scotch tape made of polytetrafluoroethylene (PTFE, 50 μm). First, the Scotch tape substrate was attached on a SiO_2_ wafer for ease of fabrication. The as-purchased Scotch tape had a large surface roughness, so a thin polyimide layer (9 μm) was spin-coated on the tape; the polyimide layer significantly reduced the roughness of Scotch tape, as shown in Fig. 3. To create gate electrodes, aluminum was evaporated using a shadow mask on the sample. Then, the gate dielectric was formed on the aluminum layer by oxidizing the surface by oxygen plasma at 12 mTorr, as described above. Gold source/drain electrodes were evaporated on the AlO_X_ gate dielectric using a shadow mask. Finally, a graphene channel with polymeric protective layers was transferred onto the top by using the water-free transfer of graphene, as described in our previous study^22^. The sample was exposed to neither solvent nor temperatures of more than 150 °C during fabrication. The device structure is shown in Fig. 1a.

First, we tested the AlO_X_ gate dielectric layer on the Scotch tape substrate. The measured capacitance was 1.5 (±0.3) μF cm^−2^, which is almost the same as that of the AlO_X_ layer made on a SiO_2_ substrate. The leakage current through the gate dielectric was an order of magnitude higher, and the breakdown voltage was approximately 0.5 V lower as compared to AlO_X_ on SiO_2_ (see Supplementary Information Fig. S1). These degraded insulating properties can be attributed to the soft substrate surface, which results in a rough bottom aluminum layer. Nonetheless, the AlO_X_ gate dielectric prepared on Scotch tape exhibited sufficient electrical performance such that it can be used in low-voltage GFETs with a negligible gate leakage. After fabricating the GFETs on the Scotch tape substrate (GFETs/Scotch), the GFETs/Scotch were separated from SiO_2_ and attached on a banknote by using the adhesive remaining on the backside of Scotch tape (Fig. 4a). The electrical performance of the samples was measured, and the field-effect mobility (μ_h_ for holes; μ_e_ for electrons) values were extracted from a diffusive transport model of GFET (see Supplementary Information Table S1 for detailed parameters)^29^. As summarized in Fig. 4a,b, the electrical performance of the GFETs/Scotch did not appreciably change after being transferred onto a banknote. Another set of samples was separated from the SiO_2_ wafer and attached on office paper. The paper was crumpled and flattened, and the electrical performance of the GFETs/Scotch was measured. As shown in Fig. 4c, GFETs/Scotch still exhibited satisfactory operation after extreme bending and showed only a slight decrease in mobility. In a previous study, a significantly thin layer of plastic foil substrate (2 μm) was used to make crumpled organic transistor arrays^30^. The notable bending property was attributed to the remarkably low foil thickness, which can sometimes cause difficulties in handling it appropriately. However, the GFETs/Scotch prepared in the present work exhibited sufficient rigidity for facile use and attachment on various objects. The electrical performance of GFETs/Scotch under three different substrate conditions is summarized in Fig. 4d (see Supplementary Information Fig. S2 for drain current vs. drain-to-source voltage curves).

Excessive surface strain induces the formation of cracks or irreversible deformation when a flexible device is bent beyond its limit, which in turn results in permanent device failure. Therefore, the range of feasible bending radii is determined by the maximum surface strain exerted on the active layer. The maximum strain at the top layer of the bent GFETs/Scotch was obtained by employing a finite element analysis with two-dimensional (2D) plain strain condition^31^. As shown in Fig. 5a, two model structures were studied: Scotch tape attached on a sheet of office paper (Fig. 4c) and a commonly used polyimide film as a control sample. When an oxidized AlO_X_/Al sample on polyimide was folded, the insulating property of AlO_X_ was completely lost, and crease marks remained. We imposed displacements on the bottom of the structures corresponding to bending radii and measured the lateral strain at the center of the top layer. In Fig. 5b, the Scotch tape sample exhibits a significantly lower surface strain compared to regular polyimide sample by a factor of five at a given bending radius. This numerical analysis can be understood in light of a 2D shear-lag model, which describes strain propagation in multilayers^32^. When the bottom layer is subject to a uniform strain ε_bottom_, the shear-lag model predicts the maximum strain at the top by Equation 2


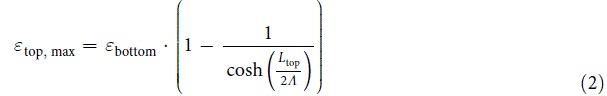


where *L*_top_ is the lateral dimension of the top layer, and Λ is a parameter proportional to the 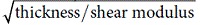 of the middle layer. The reduced strain in the Scotch tape sample is attributed to its soft silicone adhesive interlayer with a relatively low shear modulus, where mechanical load is not effectively transferred (see Fig. 5c,d). Therefore, the GFETs/Scotch on the office paper can be crumpled with a high degree of bending even though the total film thickness is greater than that of a typical polyimide sample. The effects of the strain-releasing adhesive layer are further corroborated by consecutive bending tests as shown in Fig. 6. A thin layer of aluminum deposited on the two substrate samples (Scotch tape attached on office paper and polyimide film) was bent and released consecutively with a bending radius of 1 mm. In Fig. 6, the aluminum layer on the Scotch tape substrate shows much less resistance increase against the mechanical strain from the bending and releasing, compared with the polyimide substrate. The less change on the resistance of the Scotch tape sample is attributed to its adhesive layer, which reduces the mechanical strain on the top.

We demonstrated flexible GFETs on a Scotch tape substrate. The GFETs/Scotch was easily laminated onto diverse nonconventional substrates, such as a pen, human skin, and a banknote, using Scotch tape adhesive. The soft nature of Scotch tape allows for the sample to be crumpled without leading to device failure. From mechanical calculations, we found that the Scotch tape adhesive with a low Young’s modulus relaxes the strain applied to the top surface when the GFETs/Scotch sample is bent. For practical low-power electronic applications, high-quality AlO_X_ was fabricated as the gate dielectric of the GFETs by oxygen plasma treatment. The AlO_X_ exhibited a negligible gate leakage current, and a supply voltage of only 2.5 V was sufficient to operate the GFETs because of its high capacitance of 1.5 μF cm^−2^. We envision that such a device concept for flexible graphene transistors on Scotch tape can be exploited and extended to ubiquitous electronic applications wherein electronics can be easily placed in any desired position.

## Methods

### Fabrication of graphene transistors on Scotch tape

Scotch tape (3M^TM^, 5480) was attached on a silicon dioxide wafer piece. A polyimide solution (VTEC^TM^, PI-1388) was coated onto the Scotch tape sample at 3000 rpm for 30 s, followed by sequential baking at 60 and 150 °C for 10 min. An aluminum layer (30 nm) as gate electrodes was deposited in a thermal evaporator with a shadow mask. The aluminum surface was oxidized in an oxygen plasma chamber with an RF power of 250 W for 7 min to form the gate dielectric layer. During plasma treatment, the oxygen pressure was set to the lowest possible value while maintaining the plasma; the lowest pressure value was 12 mTorr in our plasma chamber. Source/drain electrodes (40-nm-thick gold on 5-nm-thick titanium) were deposited in a thermal evaporator using a shadow mask. Finally, a graphene channel with polymeric protective bilayers was laminated on the top of the sample by employing the water-free transfer method as described in our previous study^22^. Poly(methyl methacrylate) and polybutadiene were used for supporting bilayers of graphene transfer and for passivation against detrimental ambient species to prevent Fermi level change^33^,^34^,^35^ and charge-impurity scattering^36^.

## Additional Information

**How to cite this article**: Chung, Y. *et al.* Ubiquitous Graphene Electronics on Scotch Tape. *Sci. Rep.* **5**, 12575; doi: 10.1038/srep12575 (2015).

## Supplementary Material

Supplementary Information

## Figures and Tables

**Figure 1:**
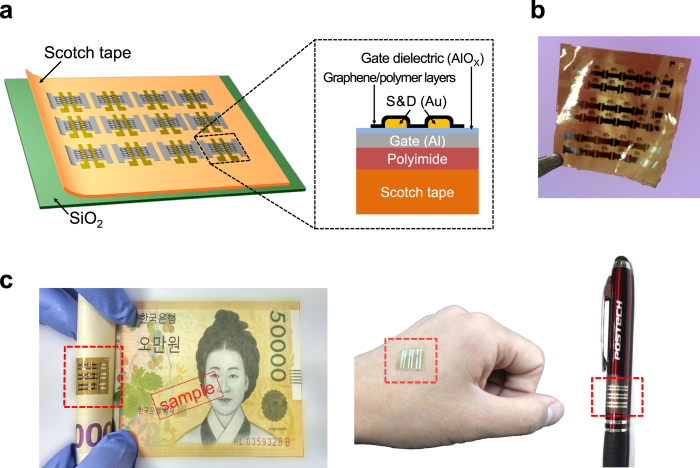
Device structure and its applications. (**a**) Schematic of device structure. The Scotch tape substrate was attached on a silicon dioxide (SiO_2_) wafer during fabrication. (**b**) After device fabrication, the Scotch tape substrate was easily peeled off from the wafer. (**c**) The Scotch tape device was then attached on nonconventional objects such as banknote (W 50,000 Korean won), human skin, and pen (Copyright 2013 Pohang University of Science and Technology).

**Figure 2:**
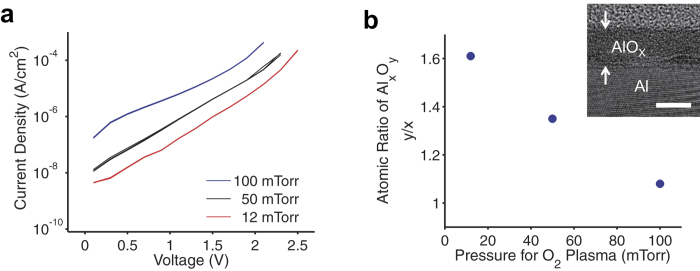
Analysis on oxidized aluminum (AlO_X_) thin film. (**a**) Leakage current of AlO_X_ under different pressure conditions during plasma treatment. As the pressure decreased, the leakage current through AlO_X_ was reduced, and the breakdown voltage increased. (**b**) X-ray photoelectron spectroscopy (XPS) data of AlO_X_ prepared under different pressure conditions. The relative ratio of oxygen to aluminum increased as the oxygen pressure during plasma treatment decreased. A higher oxygen ratio in AlO_X_ resulted in a better insulating property. Inset shows the cross-sectional transmission electron microscopy image of AlO_X_ prepared by oxygen plasma treatment on a thin aluminum film at 12 mTorr. The thickness of the AlOx layer was estimated to be 5.7 nm. Scale bar, 5 nm.

**Figure 3:**
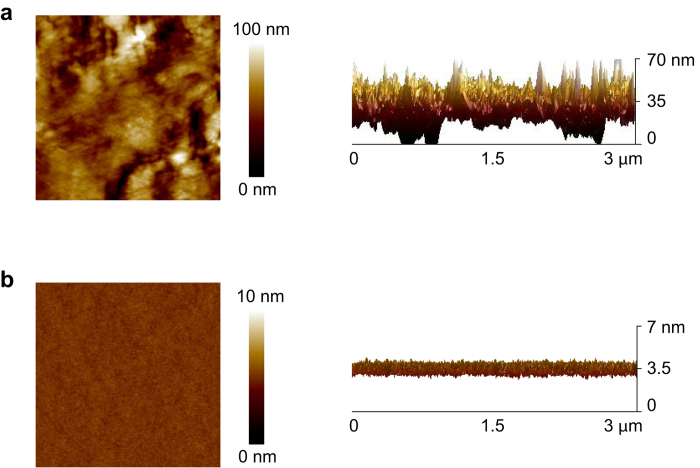
Height profile of Scotch tape surface measured by atomic force microscopy. The scan size is 3 × 3 μm. (**a**) As-purchased Scotch tape exhibited considerably high surface roughness. Root-mean-square roughness: 28.1 nm. (**b**) After spin coating of a thin layer of polyimide (9 μm) on the Scotch tape, the surface roughness was significantly reduced to be similar as silicon wafer. Root-mean-square roughness: 0.225 nm.

**Figure 4:**
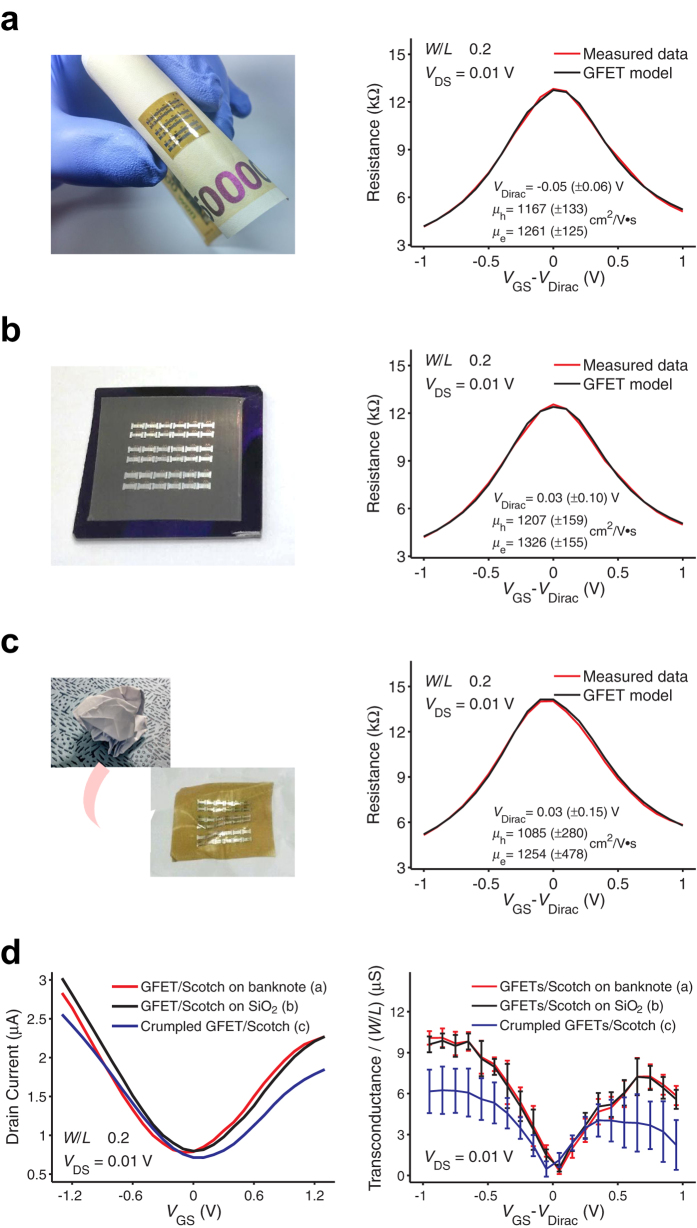
Electrical measurement data of graphene field-effect transistors on Scotch tape (GFETs/Scotch). The channel width was fixed to 85 μm, and the width-to-length ratio (*W*/*L*) values were 0.2 and 0.45. (**a**–**c**) Transistor channel resistance vs. gate-to-source voltage curves for GFETs/Scotch attached on different substrates. (**a**) GFETs/Scotch samples were attached on a banknote. While the samples were bent with a bending radius of 0.5 cm, the electrical properties remained the same (the maximum surface strain was less than 1%, see Fig. 5). (**b**) As-fabricated GFETs/Scotch on a SiO_2_ wafer. (**c**) The GFETs/Scotch on a paper were crumpled, flattened, and measurements were performed. (**d**) Performance summary of the GFETs/Scotch on the three different substrates. All measurements were performed in ambient air, and more than 10 devices were measured for each sample set.

**Figure 5:**
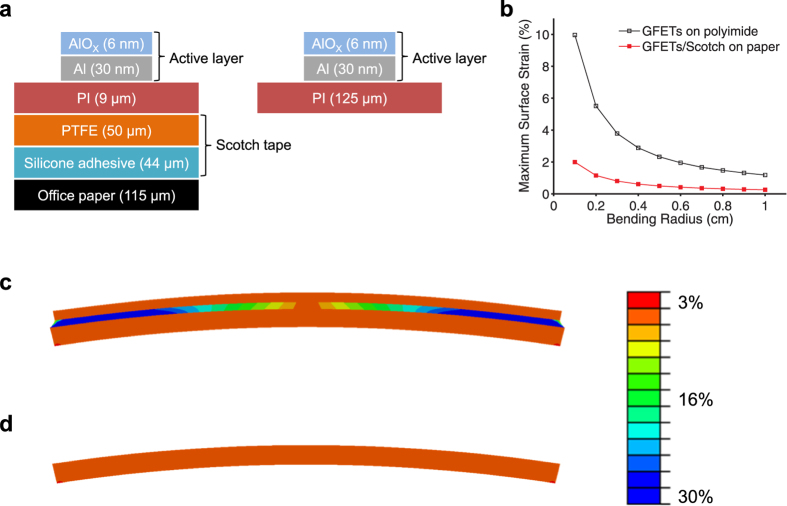
Finite element analysis on graphene transistors on Scotch tape. (**a**) Model structure for surface stain calculation [left: GFETs/Scotch on office paper; right: GFETs on a polyimide (PI) film]. (**b**) Surface strain data as a function of bending radius. Although the thickness of the GFETs/Scotch on the office paper is approximately twice that of the GFETs on polyimide, the surface strain on the GFETs/Scotch is five times lower. This result is attributed to the soft silicon adhesive interlayer, which relaxes the strain applied to the top surface. (**c**,**d**) Assuming the model structure in (**a**) is bent with a bending radius of 0.5 cm, the distribution of shear strain is calculated by using a finite element analysis. (**c**) GFETs/Scotch on office paper. The shear strain is concentrated in the silicone adhesive interlayer where the shear modulus is the lowest. (**d**) GFETs on a polyimide film. The shear strain is uniformly distributed.

**Figure 6:**
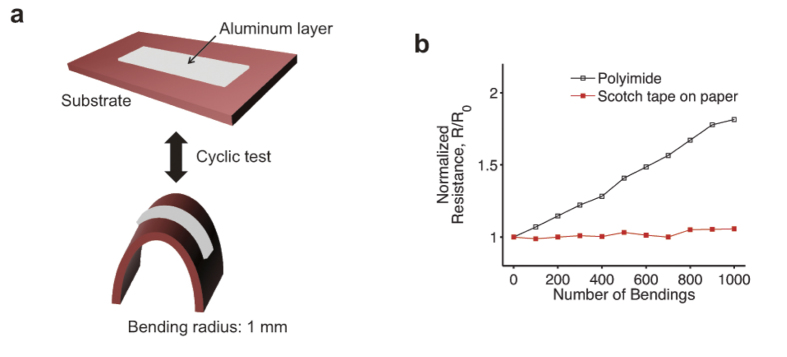
Resistance change of metal thin film against consecutive mechanical strain. (**a**) Schematic of device structure and experiment. A thin layer of aluminum (thickness: 300 nm; length: 2 cm; width: 0.2 cm) was deposited on a Scotch tape attached on office paper and a polyimide film. Detailed substrate structure is shown in Fig. 5(a). (**b**) Normalized resistance data of the aluminum layer as a function of the number of bendings. Electrical measurements were performed while the sample was flat. As explained in Fig. 5, the adhesive layer of the Scotch tape relaxes the strain applied to the top; therefore, the aluminum layer on Scotch tape exhibits much less increase on its resistance value against mechanical strain than the aluminum on the regular polyimide substrate.
