# Supplementary material for: Ubiquitous Graphene Electronics on Scotch Tape
Source: Sci Rep. 2015 Jul 29;5:12575. doi: 10.1038/srep12575 (PMC4649894; doi:10.1038/srep12575)
Supplement: Supplementary Information [file srep12575-s1.doc]

**Supplementary Information**

Ubiquitous Graphene Electronics on Scotch Tape

Yoonyoung Chung^†^, Hyun Ho Kim^†^, Sangryun Lee, Eunho Lee, Seong Won Kim,

Seunghwa Ryu, and Kilwon Cho*

^†^ These authors contributed equally to this work.


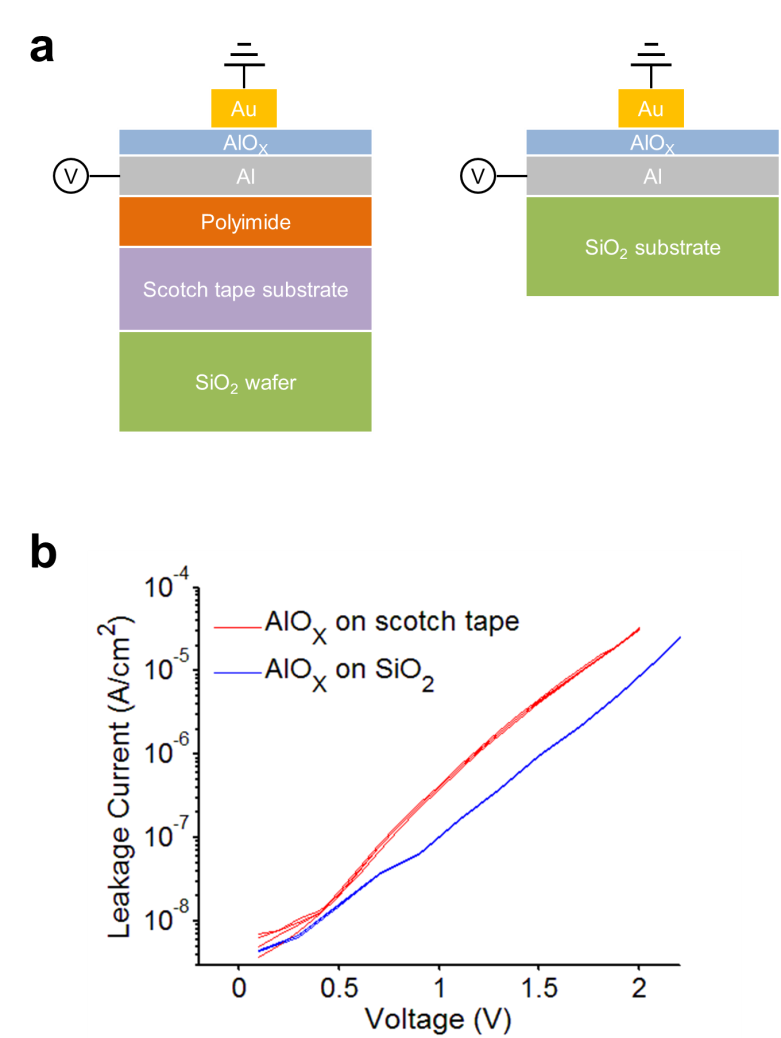


Figure S1 | Leakage current through aluminum oxide (AlO_X_) made on Scotch tape and silicon dioxide (SiO_2_) substrates. (a) Schematic of the device structure. (b) Current data between the two samples. The AlO_X_ layer on Scotch tape has an order of magnitude higher leakage than the AlO_X_ on SiO_2_.


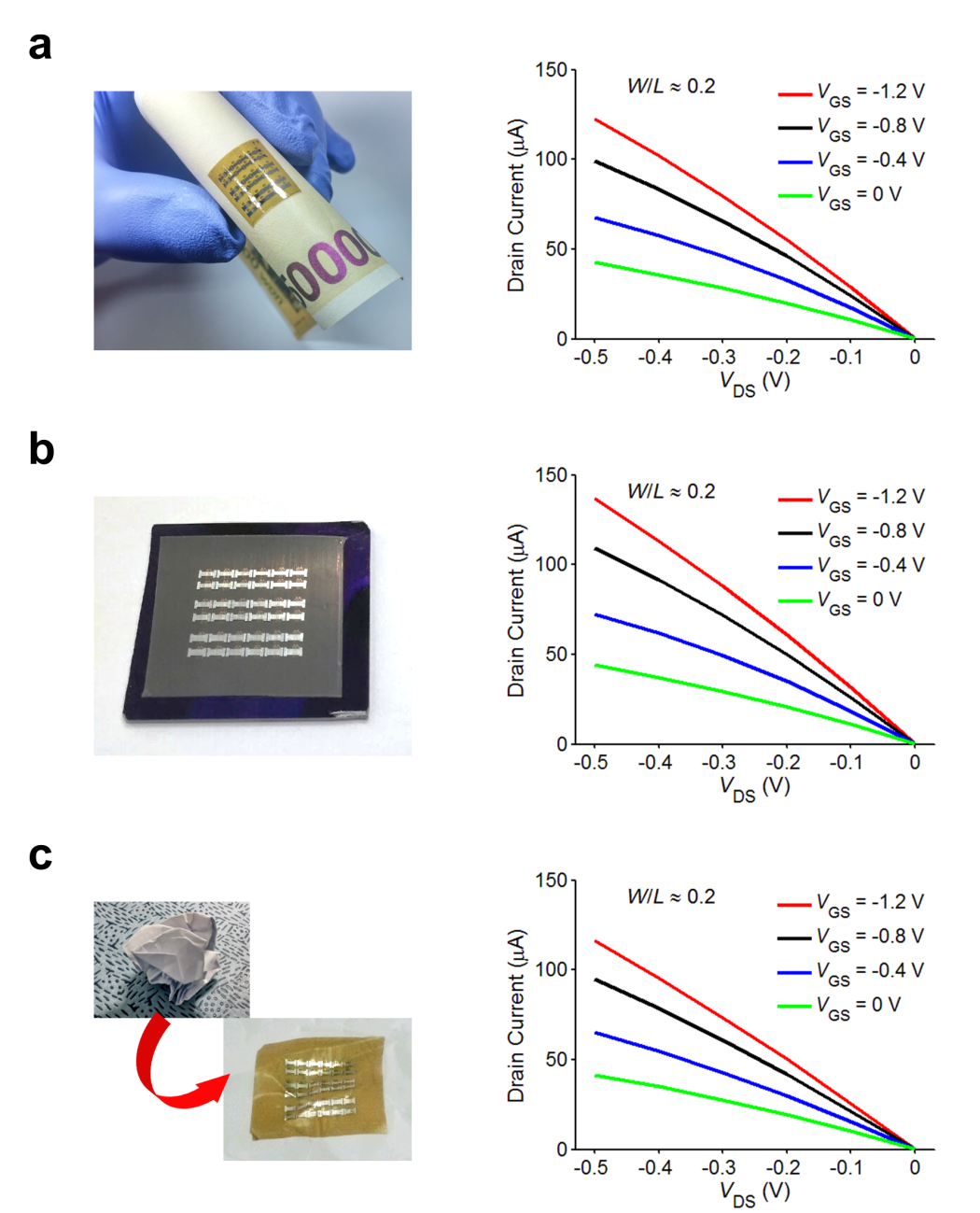


Figure S2 | Drain current vs. drain-to-source voltage curves of graphene field-effect transistors on Scotch tape (GFETs/Scotch) attached on different substrates. (a) GFETs/Scotch samples were attached on a banknote. (b) As-fabricated GFETs/Scotch on a SiO_2_ wafer. (c) The GFETs/Scotch on a paper were crumpled, flattened, and measurements were performed. All measurements were performed in ambient air.

|  | **GFETs/Scotch on SiO_2_ wafer** | **GFETs/Scotch on banknote** | **Crumpled GFETs/Scotch** |
| --- | --- | --- | --- |
| ***V*_Dirac_ (V)** | 0.03 (±0.10) | -0.05 (±0.06) | 0.03 (±0.10) |
| ***μ* _hole_ (cm^2^/V∙s)** | 1206.8 (±159.0) | 1167.0 (±132.6) | 1085.0 (±280.5) |
| ***R*_contact,h_ (ohm)** | 514.5 (±241.2) | 383.8 (±211.4) | 2767.7 (±456.5) |
| ***μ* _electron_ (cm^2^/V∙s)** | 1326.3 (±155.0) | 1261.0 (±125.4) | 1254.0 (±478.2) |
| ***R*_contact,e_ (ohm)** | 1656.8 (±687.9) | 1667.5 (±720.3) | 2411.0 (±1014.3) |
| ***n*_0_ (cm^-2^)** | 2.17 (±0.24)× 10^12^ | 2.20 (±0.19) ×10^12^ | 1.87 (±0.31) ×10^12^ |

Table S1 | Transistor performance parameters extracted by using the GFET model described in Ref. 29.
